# Prevention and Control of Multidrug-Resistant Bacteria in The Netherlands and Germany—The Impact of Healthcare Structures

**DOI:** 10.3390/ijerph17072337

**Published:** 2020-03-30

**Authors:** Robin Köck, Karsten Becker, Evgeny A. Idelevich, Annette Jurke, Corinna Glasner, Ron Hendrix, Alexander W. Friedrich

**Affiliations:** 1 Institute of Hygiene, DRK Kliniken Berlin, Spandauer Damm 130, 14050 Berlin, Germany; 2 Friedrich-Loeffler-Institute for Medical Microbiology, University of Greifswald, Ferdinand-Sauerbruch-Straße, 17475 Greifswald, Germany; Karsten.Becker@med.uni-greifswald.de; 3 Institute of Medical Microbiology, University Hospital Münster, Domagkstraße 10, 48149 Münster, Germany; evgeny.idelevich@ukmuenster.de; 4 Landeszentrum Gesundheit NRW, Gesundheitscampus 10, 44801 Bochum, Germany; Annette.Jurke@lzg.nrw.de; 5 Department of Medical Microbiology and Infection Prevention, University of Groningen, University Medical Center Groningen, Hanzeplein 1, 9700RB Groningen, The Netherlands; c.glasner@umcg.nl (C.G.); m.g.r.hendrix@umcg.nl (R.H.)

**Keywords:** antimicrobial resistance, Euregio, MRSA, ESBL, MDRO, infection prevention, antibiotic use

## Abstract

The Netherlands and Germany are neighbouring countries within the European Union but are differently affected by multidrug-resistant microorganisms (MDRO). In this narrative review, we summarize data about antibiotic use, the occurrence of MDRO and healthcare-associated infections in these two countries, as well as data about organizational and structural differences between the Dutch and German healthcare systems. These results are discussed with a focus on whether or how the organization of healthcare influences MDRO prevention. We found that from the point of view of MDRO prevention, a higher density of inpatient care, a higher number of hospitals, a longer length of stay and lower staffing ratios might facilitate MDRO dissemination in German hospitals.

## 1. Introduction

The Netherlands and Germany are neighbouring countries in the European Union sharing a 567 km border. The two countries are strongly interconnected on a historical, cultural, economic, legislative and social level. However, from the perspective of infection prevention and control (IPC) specialists, there are major differences between these two countries when comparing rates of multidrug-resistant microorganisms (MDRO). The question is how these differences can be explained.

**Figure 1:**
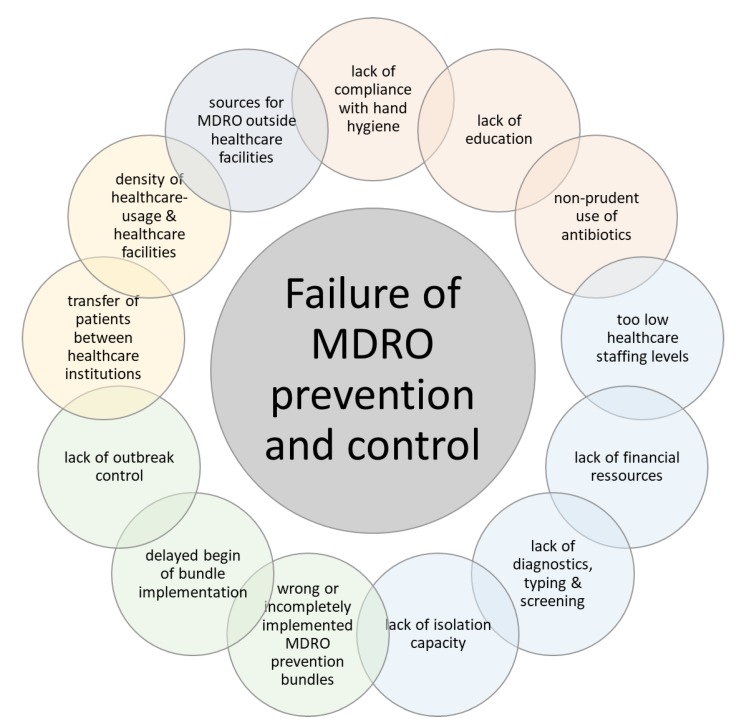
Typical reasons for the failure of infection and control of multidrug-resistant microorganisms (MDRO).

Typical reasons for the failure of MDRO-related IPC measures are shown in Figure 1. Many of these factors are structural aspects, which have their roots in the structures of different healthcare systems, and which are hard to influence by IPC experts in single hospitals. In addition, sources of MDRO outside the healthcare system might also impact the dissemination of these pathogens.

Hence, in this narrative review we aimed to (1) describe the differences in the occurrence of MDRO, healthcare-associated infections (HAI) and antibiotic use between The Netherlands and Germany; (2) summarize data elucidating the structure the healthcare systems in these two countries and (3) to interpret both data from the point of view of IPC specialists.

## 2. Multidrug-Resistant Microorganisms, Antibiotic Use and Healthcare-Associated Infections in The Netherlands and Germany

### 2.1. Data about the Occurrence of MDRO

**Figure 2:**
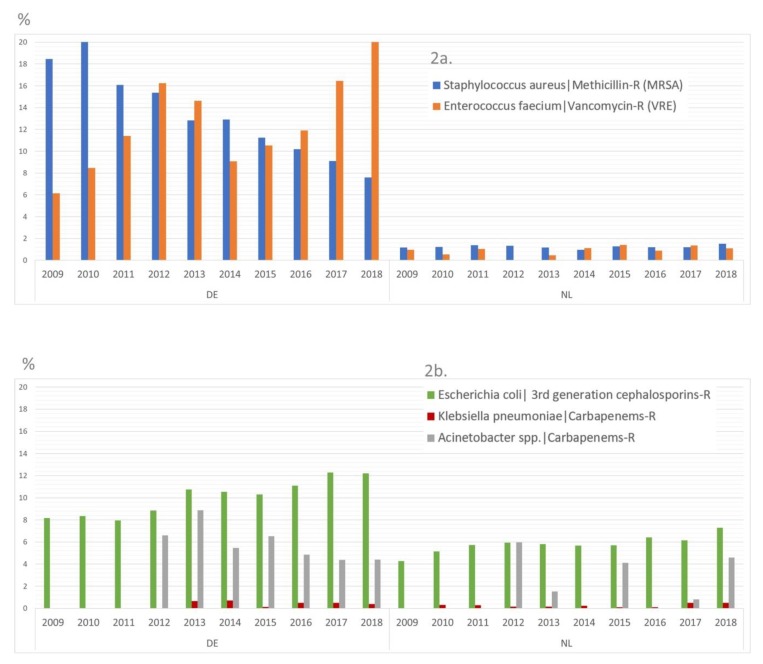
Percentage of resistant isolates from bloodstream infections according to EARS-Net data 2009-2018. (**a**) for methicillin-resistant *Staphylococcus aureus* (MRSA) and vancomycin-resistant *Enterococcus faecium* (VRE); (**b**) for third-generation cephalosporin-resistant *Escherichia coli,* carbapenem-resistant *Klebsiella pneumoniae* and carbapenem-resistant *Acinetobacter* spp.

Data from the European Antimicrobial Resistance Surveillance Network (EARS-Net) on the proportion of MDRO among clinically relevant pathogens isolated from invasive infections indicate that, overall, Germany seems to have a bigger challenge than The Netherlands (Figure 2). In consequence, a recent study estimated that the burden of MDRO-infections described by the numbers, deaths, incidences and mortality rates are also divergent (Table 1, [1]).

### 2.2. Data about HAI

The rate of HAI was recently assessed in an EU-wide study: in Germany, the prevalence of nosocomial infections was 5% (3.8–67, n = 516 infections, 46 participating hospitals) vs. 7.4% in The Netherlands (6.2–8.8, n = 598 infections in 33 participating hospitals) [2]. However, a continuous surveillance system established in The Netherlands yielded different results: in 33 hospitals, the overall prevalence of HAI was 4.6% in 2018 [3].

In 2018, the incidence of *Clostridioides difficile*-associated infections (CDI) was 3.17/10,000 patient-days in 24 Dutch hospitals participating in a surveillance system compared with 5.47/10,000 patient-days in 571 German hospitals [4,5]. The locations of the onset were healthcare facilities in 54% of the cases on the Dutch and 56% on the German side of the border [4,5].

The use of diagnostics might also affect HAI rates. A recent study evaluated the quality of microbiological diagnostics of bloodstream infections among European countries [6]. According to this study, 21.6 blood culture (BC) sets per 100 hospitalizations were sampled in German (n = 10 laboratories) hospitals. Due to the relatively small number of participating laboratories from The Netherlands (n = 6), the respective Dutch data were not analyzed in the original publication. Re-assessing these data, 33 BC sets per 100 hospitalizations were sampled in The Netherlands (personal communication, E. A. Idelevich, Münster and K. Becker, Greifswald). In the overall analysis of 162 laboratories from all countries included in the survey, 18.9 BC sets per 100 hospitalisations were taken.

### 2.3. Data about Antibiotic Use

In 2017, the use of antibiotics among outpatients (in primary care) was 10.1 defined daily doses (DDD) per 1000 inhabitants in The Netherlands vs. 13.7/1000 in Germany [7]; i.e., about 33% higher. Prescribing data showed that different prescribing habits were observed especially for the group of other betalactam antibiotics, i.e., predominantly oral cefuroxime (Table 2).

**Figure 3:**
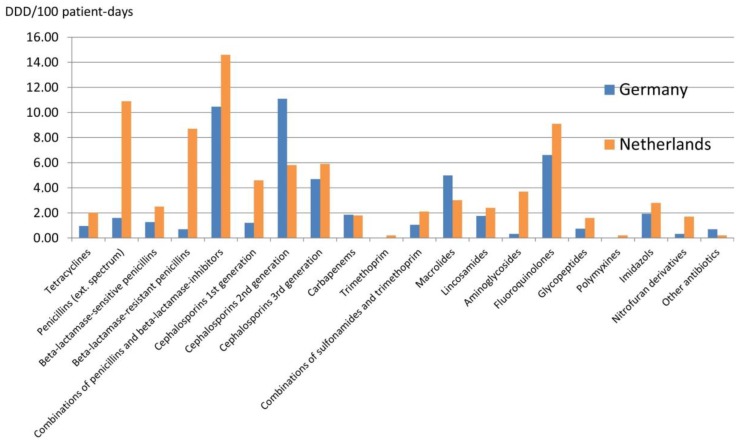
Antibiotic use in defined daily doses (DDD) per 100 patient-days for antibiotics belonging to the J01 category according to the ATCC classification system [8].

In 2016, both national surveillance systems described that the density of antibiotic use in hospitals was 84 DDD/100 patient-days in The Netherlands (n = 62 participating hospitals; with large variation for general hospitals; overall use in general hospitals was 82.5 DDD/100 patient-days and in university hospitals 92.5 DDD/100 patient-days) [8] vs. 52.5 DDD/100 patient-days in Germany (n = 148 participating hospitals; in larger and maximum care hospitals use was 57.3 DDD/100 patient-days) [9]. Qualitatively, the densities for the use of penicillins, first and third generation cephalosporins, aminoglycosides, glycopeptides and fluoroquinolones were higher in Dutch hospitals, while the densities of use for second generation cephalosporins and macrolides were lower compared with German hospitals (Figure 3).

## 3. Data about Healthcare Structures in The Netherlands and Germany

Table 3 compares data about the numbers of hospitals, the density of hospital-use, as well as staffing levels in German and Dutch hospitals.

## 4. Discussion

In this review, we tried to put the occurrence of MDRO in relation to existing data about healthcare structures in The Netherlands and Germany. Table 3 shows that the financial resources used in the Dutch and German healthcare systems are similar. However, the density of inpatient-care is much higher in Germany, as indicated by the facts that there are 2.2-times more hospital beds and 2.4-times more discharges per population. In addition, the rate of ambulatory surgery is lower for many elective interventions and the rates of hospital-days per 1000 inhabitants as well as the average length of stay are higher in Germany. At the same time, the density of hospital personnel (i.e., the staffing level per 100 hospital days) is 3.5-times higher in hospitals in The Netherlands, especially for nursing staff.

Hence, from an IPC perspective, German hospitals are clearly challenged by more patients, who are hospitalized for a longer period, but cared for by fewer personnel. Overall, the effects of these structures remain controversial and highly speculative. However, it is clear that these structures have a major impact on several aspects related to MDRO prevention:

1. Hospitalization (number of cases, length of stay) per se increases the risk of MDRO transmission (from patient-to-patient, via healthcare workers or via surfaces). Moreover, due to the higher number of hospitals in Germany, many hospitals are less specialized. Consequently, there is more patient traffic between different hospitals (as patients are sent from basic care to more specialized hospitals). In this setting, each admission bears the possibility of inter-institutional transmission, facilitating regional dissemination of MRDO. Some studies have demonstrated that this led to a clonal expansion of some MDRO [13,14].
2. Hand hygiene must be performed more rigorously in German hospitals. Due to fewer personnel and more patients, there are more contacts per individual healthcare worker. In addition, one healthcare worker has contact with more patients. If it is assumed that the compliance level with hand hygiene is similar in The Netherlands and Germany, these underlying structures might facilitate the transmission of MDRO. Moreover, a high awareness about the AMR problem among healthcare workers has been recently shown for both countries [15].
3. Although the density of hospital beds is higher in Germany, the bed occupancy rate is also higher than in The Netherlands. While in German hospitals occupancy rates are regularly above 80%, they are usually around 60%-70% in Dutch hospitals (Table 3). This has an impact on the availability of single rooms for isolation. Besides the rather low scientific evidence for the isolation of patients with MDRO and the lack of randomized trials regarding this issue [16], as well as the potential disadvantages of “isolated care” in single-rooms (e.g., stigmatization and less awareness), many clinicians and hospital administrations in Germany refer to a lack of isolation beds and financial penalties due to blocked beds, when arguing against single-room isolation. In The Netherlands, due to the structural difference, single-room isolation is easier to organize and rather undisputed for most MDROs including methicillin-resistant *Staphylococcus aureus*, vancomycin-resistant *enterococci*, multidrug-resistant Gram-negative bacteria (including even extended-spectrum betalactamase-producing, carbapenem-susceptible *Enterbacteriaceae*). For risk patients, the concept of pre-emptive isolation at admission as long as screening results are pending, is also a key component of Dutch IPC recommendations. In Germany, pre-emptive isolation is performed for patients with a high risk of carriage of carbapenem-resistant *Enterobacteriaceae* or *Acinetobacter baumannii* or for patients with a very high risk of MRSA carriage (e.g., known carriage from previous hospitalizations). However, for MRSA, the fact that >30% of patients at admission are risk patients in Germany, prevents the ability to use pre-emptive isolation for all patients at risk [17].

However, a causal relationship between healthcare structures and prevalence of MDRO is rather difficult to prove. This is partly because of the available contradictory data. For example, the prevalence of HAI, such as urinary-tract infections or catheter-related bloodstream infections, seem to be similar in the two countries [2,3]. For certain surgical site infections, up to three times more infections have been described in The Netherlands compared to Germany [2]. This seems in contrast to the theory that more staff facilitate hand hygiene compliance, as hand disinfection is regarded as one of the cornerstones of HAI prevention. Hence, as we are not aware of any structured investigations assessing hand hygiene indications and compliance rates in the border region, such assessments should be stimulated. However, on a population level, the higher density of care in Germany (more hospitalizations, more hospital-days per patient) vs. the Dutch healthcare system characterized by waiting lists for certain elective procedures and stricter prioritization, might increase the rate of HAIs per inhabitants in Germany. Clear data is lacking regarding this issue.

The prudent use of antibiotics is a public health priority. In The Netherlands, antibiotic use among outpatients is about 30% lower than in Germany. The reasons have not been studied in detail, but could involve both educational and socio-cultural aspects (e.g., attitude towards prescribing antibiotics, expectations of patients etc.) [18,19]. One example is the dissimilar use of oral cefuroxime in these two countries (Table 2). Oral cefuroxime has a lower resorption when given orally and a rather high selection pressure for MDRO. In a study, performed in the Dutch—German border region it was demonstrated that the proportion of children in ambulatory care, who received at least one antibiotic within a one-year period was 29.8% in The Netherlands and 38.9% in Germany. In this study, oral cefuroxime accounted for 25% of the prescriptions in Germany and 0.1% in The Netherlands [20].

While the data for outpatients clearly indicate less selection pressure in The Netherlands and are a good argument for less MDRO, we found that the density of antibiotic use measured in DDD/100 patient-days in Dutch hospitals is higher than in German hospitals (Figure 3). This is true for general hospitals as well as larger teaching hospitals. We are unsure to what extent this is a virtual difference, which could be explained by the shorter length of stay in Dutch hospitals (when using patient-days as the denominator) or by the more extensive use of penicillins in Dutch hospitals (when actually prescribed doses of penicillins are much higher than the WHO-defined DDD). On the other hand, the more frequent use of 3rd generation cephalosporins seems reasonable, as selective digestive decontamination for pneumonia-prevention is rarely performed in German, but often in Dutch hospitals. Moreover, for fluoroquinolones, the DDD describes the actual daily dose used in clinical care more accurately than for penicillins. Hence, the reason for the substantially higher use of fluoroquinolones in Dutch hospitals remains unclear. Overall, these discrepancies point out that studies assessing the antibiotic use in Dutch and German hospitals in a more detailed manner (e.g., on wards treating matched patient-collectives such as intensive care units) should be inspired. This should also help to explain why despite the higher density of antibiotic use, data about the occurrence of nosocomial *C. difficile*-associated infections indicate that antibiotic selection pressure might be lower in Dutch hospitals.

Assessing the number of blood culture sets (BCs) related to patient-days could be a quality indicator for microbiological diagnostics. More BCs are sampled in Netherlands than in Germany, but for both countries, BC rates were higher than the average for European countries [6]. However, the interpretability of this parameter when assessed for complete hospitals might be very limited, if the types of hospitals and hospital-departments included in the analysis differ or are even undefined. Representative data that would allow for a more robust comparison, such as BC rates among patients on intensive care units, are currently available only for German ICUs indicating a high rate of diagnostics (mean 170 BC sets/1000 patients-days, [21]).

However, formally it is a clear advantage that in The Netherlands there are mandatory regulations for antibiotic and diagnostic stewardship staff including microbiologists, infectious disease specialists and pharmacists. Such regulations are currently on the way in Germany, but respective scientific recommendations have not reached the level of legal liability.

In both countries, sources for MDRO outside healthcare facilities have been studied extensively. It was found that regionally livestock-associated MRSA play a major role for colonization and infections for those persons who are directly exposed to livestock [22,23]. In addition, ESBL-producing *Enterobacteriaceae* (ESBL-E) from food items have been demonstrated to be responsible for only a minority of ESBL-E infections among humans [24,25]. Antibiotic use among livestock was markedly reduced (by more than 50%) in both countries [26]. Hence, there is no evidence that there are major differences in the control of sources for MDRO outside healthcare facilities. However, the differences in outpatient antibiotic use mentioned above may contribute to more selection pressure and a higher probability for the emergence of MDRO in the community in Germany.

Overall, one major limitation should be mentioned. While for some indicators, such as antibiotic resistance rates or antibiotic use among outpatients, data are available from international surveillance networks using a defined technique, other indicators used in this review are from national surveillance programs or studies. Of course, both of these different data sources might cause biases when comparing The Netherlands and Germany (e.g., different representativeness of the data source for the country, slightly different assessment techniques). However, we carefully checked the included sources and pointed out where data sources might have caused a major bias.

## 5. Conclusions

Although regional IPC measures have been proven as a promising strategy [27,28], the various healthcare structural aspects that can hardly be influenced by local IPC specialists must also be recognized. Those IPC factors include differences of reimbursement systems, staffing levels, hospital or healthcare densities and antibiotic use between The Netherlands and Germany.

## Figures and Tables

**Table 1:** Estimated median number of cases (incidences per 100,000 inhabitants) and median numbers of deaths (mortality per 100,000 inhabitants) for infections due to MDRO in 2015 [1].

Pathogen *	Germany		The Netherlands	
	*Cases* *(Incidence)*	*Deaths* *(Mortality)*	*Cases* *(Incidence)*	*Deaths* *(Mortality)*
CRAB	278 (0.34)	24 (0.03)	14 (0.08)	1 (0.01)
VRE	3089 (3.80)	206 (0.25)	63 (0.37)	4 (0.02)
CRKP	125 (0.15)	3 (0)	0 (0)	0 (0)
MRSA	13,684 (16.85)	653 (0.80)	249 (1.47)	12 (0.07)
3rd generation-cephalosporin-res. *E. coli*	28,392 (34.97)	868 (1.07)	3503 (20.73)	107 (0.63)
all assessed MDROs	54,509 (67.13)	2363 (2.91)	4982 (29.48)	206 (1.22)

* Methicillin-resistant *Staphylococcus aureus* (MRSA), vancomycin-resistant *Enterococcus faecium* (VRE), carbapenem-resistant *Klebsiella pneumoniae* (CRKP) and carbapenem-resistant *Acinetobacter baumannii* (CRAB).

**Table 2:** Antibiotic use among outpatients in defined daily doses (DDD) per 1000 inhabitants per day in 2017 [7].

Type of Antibiotic	EU-Mean	Germany	The Netherlands
Total use	21.8	13.7	10.1
Penicillins	11.5	5.0	4.0
Other betalactams	2.0	2.8	0
Sulfonamide/trimethoprim	0.6	0.5	0.4
Macrolides	2.9	2.1	1.4
Tetracyclines	2.2	1.8	2.0
Quinolones	1.6	1.1	0.7
Other antibiotics	1.1	0.5	1.5

**Table 3:** Structures of the healthcare systems in The Netherlands and Germany [10,11].

Indicator		The Netherlands	Germany
Population		16,979,140	82,175,684
Health expenditures in Mio. €		72,788.63	351,701.00
	Pro inhabitant in €	3,885	4,160
Life expectancy at birth (years)		81.7	81.0
Length of hospital stay (curative care) in days		5.0	7.5
Hospital days per year		8,268165	146,048,193
	per 1,000 inhabitants	486.96	1777.27
Hospital beds total (curative, rehabilitative, long-term care)		61,767	663,941
	Curative	51,176	498,718
	rehabilitative	1946	165,223
	per 1000 inhabitants	3.64	8.08
	Hospital beds/100,000 inhabitants curative care	301	606
Bed occupancy rate (curative care)		66%	80%
Hospital discharges		1,649,905	19,480,503
	Per 1000 inhabitants	97.17	237.06
Hospital personnel (full-time equivalents)		198,670	988,000
	per 100 patient-days	2.40	0.68
Physicians		21,808	166,000
	per 100 patient-days	0.26	0.11
Qualified nurses/midwives		58,489	341,000
	per 100 patient-days	0.71	0.23
Nursing associates		11,563	34,000
	per 100 patient-days	0.14	0.02
Antibiotic Stewardship Teams			
		1 team * per hospital mandatorily	1 FTE per 500 beds recommended *
Selected interventions per 100,000 inhabitants			
	cataract-surgery	1014	1041
	appendectomies	96	155
	Transluminal coronary angioplasty (PTCA)	234	406
% of 1-day interventions (day-patients, outpatients)			
	cataract-surgery	99.6%	82.5%
	tonsillectomies	68.4%	4.0%
	Inguinal hernia	80.2%	0.3%

* In NL: Including medical microbiologist, infectious disease specialist, pharmacist; in Germany: FTE = full-time equivalent (additionally further FTEs for special departments); recommended in antibiotic stewardship guideline [12], but not mandatory for all hospitals. On the level of German federal states, some states start setting mandatory standards.
